# Three-dimensional printing models of horseshoe kidney and duplicated pelvicalyceal collecting system for flexible ureteroscopy training: a pilot study

**DOI:** 10.1590/S1677-5538.IBJU.2021.99.10

**Published:** 2021-03-05

**Authors:** Ulisses L. G. Pereira, José R. P. Albero, Marina L. P. Becalli, Francisco J. B. Sampaio, Luciano A. Favorito

**Affiliations:** 1 Universidade do Estado do Rio de Janeiro Unidade de Pesquisa Urogenital Rio de JaneiroRJ Brasil Unidade de Pesquisa Urogenital, Universidade do Estado do Rio de Janeiro – UERJ, Rio de Janeiro, RJ, Brasil.; 2 Hospital Municipal de Itamaraju ItamarajuBA Brasil Hospital Municipal de Itamaraju, Itamaraju, BA, Brasil.; 3 Universidade Federal do Sul da Bahia ItabunaBA Brasil Universidade Federal do Sul da Bahia – UFSB, Itabuna, BA, Brasil.

Congenital anomalies of the upper urinary tract comprise a diversity of abnormalities, including aberrant location, orientation and shape of the kidney, as well as aberrations of the collecting system and blood supply. Horseshoe kidney is the most common renal fusion anomaly, with prevalence of 0.25% of the population (1, 2). Ureteral anomalies of number are also frequent, particularly ureteral duplications, which have incidence of around 1/150 live births (3). The incidence of nephrolithiasis in horseshoe kidney patients is approximately 20% (4).

The anatomical properties of anomalous kidneys present substantial obstacles to endourologic procedures, especially because of the position of the renal calyces (4). Flexible ureteroscopy is a common procedure nowadays, and most training programs use virtual reality simulators. Endourologic training using simulation is very important to junior doctors because they have multiple attempts and opportunity for trial and error learning. In this study, we demonstrate a new option for endourological training, using three-dimensional (3D) printing models of kidneys with macroscopic congenital anomalies (horseshoe and duplicated pelvicalyceal collecting system).

**Figure 1:**
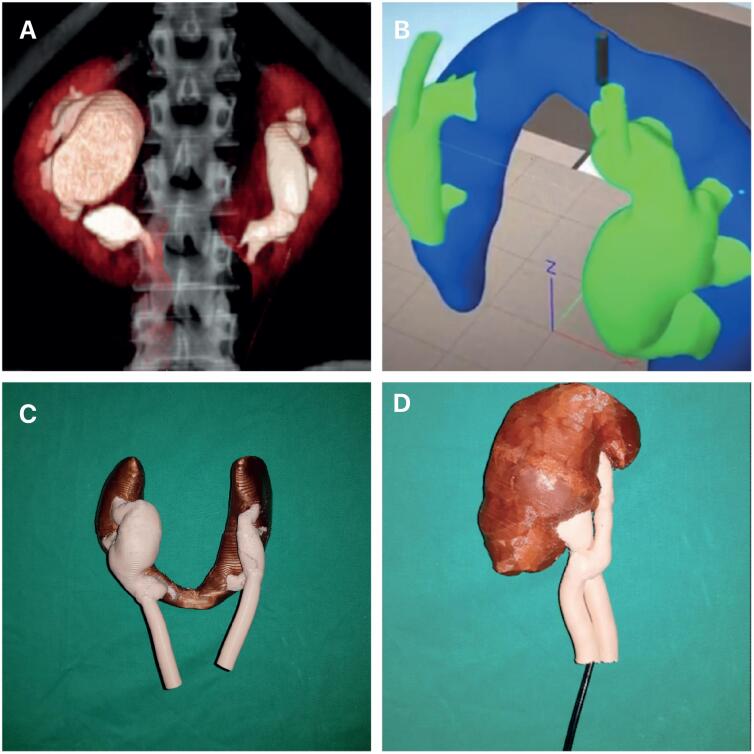
A) The figure shows the computed tomography (CT) scan image of one of the horseshoe kidneys analyzed. B) The CT image with the digital imaging and communications in medicine format was processed with the Simplify3D^®^ software and the kidneys were printed using a Flashforge Dreamer^®^ 2018 printer. C) This figure shows the 3D printed model of a horseshoe kidney. The model was made of polylactic acid and acrylonitrile butadiene styrene with diameter of 1.75 millimeters; and D) This figure shows the navigation with a digital flexible ureteroscope (3.3 x 670 mm, model DR030670, Endomaster^®^) in a 3D printed model of a 3D printed ureteral duplication model.

The study was approved according to the ethical standards of the hospital's institutional committee on experimentation with human beings (IRB: 1.171.286, CAAE: 70623417.7.0000.5259). Usable data of two patients with horseshoe kidneys and two patients with complete ureteral duplication were obtained from computed tomography (CT) scans as Digital Imaging and Communications in Medicine (DICOM) format from a public health unit (Figure-1A). These DICOM images were processed with the Simplify3D^®^ software and were printed using a Flashforge Dreamer^®^ 2018 printer (Figure-1B). The models were made of polylactic acid (PLA) and acrylonitrile butadiene styrene (ABS) with diameter of 1.75 millimeters (Figure-1C). The navigation was obtained by the same observer with a digital flexible ureteroscope (3.3 x 670 mm, model DR030670, Endomaster^®^) (Figure-1D). The 3D printed kidney has low manufacturing cost (about US$ 100), and it is relatively quick to make (on average 22 hours). The digital flexible ureteroscope could be inserted into all molds and the entire collecting system (including the lower pole and perpendicular minor calices) could be examined in horseshoe kidneys and ureteral duplications without difficulties.

Recently, the use of 3D silicone molds (cavities) of the collecting system using polyester resin endocasts was proposed for flexible ureteroscopy surgical training (5). Using polyester resin endocasts models is an interesting and inexpensive technique but is very laborious and the molds made are not as perfect as the 3D printed kidney models.

The use of 3D printed kidney models before endourological procedures for pre-surgical training is feasible and can be done with low-cost materials. The surgeon can train before proposing the appropriate surgical schedule to the patient using the 3D printed kidney.
